# Temperature, Salinity and Garlic Additive Shape the Microbial Community during Traditional Beetroot Fermentation Process

**DOI:** 10.3390/foods12163079

**Published:** 2023-08-16

**Authors:** Justyna Staninska-Pięta, Jakub Czarny, Łukasz Wolko, Paweł Cyplik, Agnieszka Drożdżyńska, Martyna Przybylak, Katarzyna Ratajczak, Agnieszka Piotrowska-Cyplik

**Affiliations:** 1 Department of Food Technology of Plant Origin, Poznan University of Life Sciences, Wojska Polskiego 31, 60-624 Poznan, Poland; 2 Institute of Forensic Genetics, Al. Mickiewicza 3/4, 85-071 Bydgoszcz, Poland; 3 Department of Biochemistry and Biotechnology, Poznan University of Life Sciences, Dojazd 11, 60-632 Poznan, Poland; 4 Department Biotechnology and Food Microbiology, Poznan University of Life Sciences, Wojska Polskiego 48, 60-627 Poznan, Poland

**Keywords:** beetroot leaven, microbiome, next generation sequencing, betalain, organic acids

## Abstract

Plant-based traditional fermented products are attracting a lot of interest in global markets. An example of them is beetroot leaven, which is valued for its high bioactive compound content. The variety of production recipes and the spontaneous nature of red beet fermentation favor its high diversity. This study aimed to analyze the impact of external factors—temperature, brine salinity, and garlic dose—on the beetroot fermentation and bacterial metapopulation responsible for this process. The research results confirmed the significant influence of the selected and analyzed factors in shaping the leaven physicochemical profile including organic acid profile and betalain content. Analysis of bacterial populations proved the crucial importance of the first 48 h of the fermentation process in establishing a stable metapopulation structure and confirmed that this is a targeted process driven by the effect of the analyzed factors. *Lactobacillaceae*, *Enterobacteriaceae*, and *Leuconostocaceae* were observed to be the core microbiome families of the fermented red beet. Regardless of the impact of the tested factors, the leaven maintained the status of a promising source of probiotic bacteria. The results of this research may be helpful in the development of the regional food sector and in improving the quality and safety of traditionally fermented products such as beetroot leaven.

## 1. Introduction

Red beet (*Beta vulgaris* L.) is widespread in Central and Eastern Europe, Asia, and America. Due to its easy cultivation and storage, which does not require large financial resources, red beet is available in European markets throughout the year [1,2]. Red beet is a good source of phenolic compounds, carotenoids, flavonoids, betalains, and many vitamins and minerals [3]. These features, together with the high average sugar content (77.5 g/L), make red beet highly useful for manufacturing fermented products of high nutritional value [2]. Beet leaven produced by spontaneous fermentation has been particularly popular in recent years on the worldwide markets, including the Polish market. Leaven is an element of Polish cultural heritage and is mentioned many times on the list of traditional products published by the Polish Ministry of Agriculture and Rural Development [4].

Despite the high popularity of beetroot leaven, a detailed metagenomic analysis of the traditional fermented beetroot’s microbiota composition has not yet been conducted. The manufacturing process is not standardized, and the recipes differ in many details such as the amount of garlic added, the brine concentration, and the processing temperature. Therefore, a large diversity of final products in the context of sensory and microbiological characteristics can be expected. As a root vegetable, beetroot is also exposed to contamination with pathogenic microorganisms constituting the soil microbiome [5]. Comprehensive research on the impact of selected parameters on red beet fermentation will allow the optimization of the manufacturing process, increase the leaven safety, and help in developing this regional food sector.

The study aimed to assess the effect of selected physicochemical parameters: temperature, sodium chloride concentration in brine, and the amount of garlic added, on spontaneous beetroot fermentation. Research focused on the qualitative and quantitative composition of microbiota supported by the analysis of selected physicochemical features.

The following research hypotheses were put forward: (1) brine salinity, temperature, and the garlic addition significantly affect the spontaneous red beet fermentation. (2) The above-mentioned factors shape the quantitative and qualitative composition of the microbiota and the properties of the final product (pH, concentration of organic acids and betalains).

## 2. Materials and Methods

### 2.1. Preparation of Red Beet Fermentation Variants

Red beets (*Beta vulgaris* L. subsp. *vulgaris* var. Demeter) were obtained from an organic farm situated in the Wielkopolska Province. Crops were fertilized with animal manure. The beetroots were rinsed with tap water (15 °C) and the leaves and hard parts (neck with crown, narrow extended taproot end) were removed. To simulate the conditions of decreased hygienic standards, procedures of careful washing and peeling were abandoned. Then, the roots were cut into 1 cm thick slices and placed randomly in sterilized glass jars with a capacity of 900 mL (400 g of beets/1 jar). Different amounts of garlic (with husks) and different concentrations of sterile brine were added to the different experimental variants. The details of the experimental variants are summarized in Table 1. The jar caps were equipped with wine tubes to prevent spillage and to maintain anaerobic conditions during the process. The fermentation was carried out for 14 days (based on the traditional recipes) in different temperature conditions (Table 1). Each experimental variant was carried out in triplicate.

### 2.2. Analysis of the Fermentation Process

The fermentation processes were analyzed based on a decrease in active acidity (as a result of microbial transformation of sugars into organic acids). Active acidity was determined electrometrically using a pH meter with a glass electrode (Elmetron, Zabrze, Poland).

### 2.3. Quantitative Microbiological Analysis of Red Beet Leaven Variants

The samples for culture-dependent quantitative microbiology analyses of the number of total culturable microorganisms (TCM) and the lactic acid bacteria (LAB) were prepared following the ISO 6887 standard. Leaven samples were taken after 48, 96, 144, 240, and 336 h of the fermentation process. The quantification of microorganisms was performed by spreading the 10-fold dilutions of the sample in growth medium (Bouillon agar (BTL, Warsaw, Poland) and MRS medium (BTL, Warsaw, Poland) for TCM and LAB, respectively). The colonies were counted according to ISO 7218 standard after 48 h of incubation at 37 °C. Plates for LAB enumeration were incubated under anaerobic conditions (AnaeroPack supplied with Oxoid™ AnaeroGen™, ThermoFisher, Waltham, MA, USA). All results are expressed as CFU/mL of red beet leaven.

### 2.4. Quantitative Microbiological Analysis—Next-Generation Sequencing

#### 2.4.1. DNA Isolation

DNA isolation from leaven samples after 14 days of fermentation was performed using the Genomic Mini kit (A&A Biotechnology, Gdańsk, Poland). The procedure is based on the instructions provided and recommended by the manufacturer. To increase the efficiency of Gram-positive cell lysis, recombinant mutanolysin (A&A Biotechnology, Gdańsk, Poland) was used. The concentration of isolates was assessed by fluorimetry method on the Qubit 3.0 device (ThermoFisher, Waltham, MA, USA) by using the dsDNA HS Assay Kit (ThermoFisher, Waltham, MA, USA). Samples within one experimental variant were pooled together after positive evaluation of their concentrations.

#### 2.4.2. PCR Amplification

For the bacteria identification, the hypervariable region V2-V9 of the bacterial 16S rRNA gene was amplified. The reaction was carried out using the Ion 16S™ Metagenomics Kit (ThermoFisher, Waltham, MA, USA) according to the manufacturer’s instructions. The reaction mixture consisted of 15 µL of Master Mix, 1.5 µL of each of the primers, and 12 µL of the DNA sample optimized to a concentration of 0.1 ng/µL. The following temperature profile was used: initial denaturation for 5 min at 95 °C; 25 cycles of: denaturation for 30 s at 95 °C, annealing for 30 s at 58 °C, extension for 20 s at 72 °C, and a final extension for 5 min at 72 °C. PCR products were purified using the Agencourt AMPure XP Reagent kit (Beckman Coulter, Brea, CA, USA), according to the manufacturer’s instructions.

#### 2.4.3. Sequencing

The sequencing procedure was described in detail in the previous publication [6]. The DNA library was constructed using the Ion Plus Fragment Library Kit (Life Technologies, Carlsbad, CA, USA) and purified using the Agencourt AMPure XP Reagent (Beckman Coulter, Brea, CA, USA) and then quantified by the Ion Universal Library Quantitation Kit and a real-time PCR instrument—Quant Studio 5 (Life Technologies, Carlsbad, CA, USA). The library was coated onto beads and sequenced using an Ion PGM System using the Ion PGM™ Hi-Q™ View Sequencing Kit on an Ion 316™ Chip Kit v2 BC (Life Technologies, Carlsbad, CA, USA).

#### 2.4.4. Bioinformatic Analysis of Results and Data Visualization

Bioinformatic analysis of the raw sequencing data was performed using CLC Genomics Workbench 20.0 with CLC Microbial Genomics Module 20.1.1 software (Qiagen, Hilden, Germany). Chimeric and low-quality reads were removed and then clustered against the SILVA v119 database at 97% similarity of operational taxonomic units (OTU) [7]. Alpha coefficients of biodiversity were determined (Chao-1 bias corrected, Simpson index). The assessment and visualization of beta-biodiversity (PCoA Bray–Curtis index and Jaccard index dendrogram clustering) were carried out using the Microbiome Analyst 2.0 Software (McGill University, Montréal, QC, Canada) [8].

### 2.5. Evaluation of the Organic Acids Profile in Beetroot Leaven

Quantitative evaluation of selected organic acids (lactic acid, acetic acid, succinic acid, L-pyroglutamic acid, formic acid, and butyric acid) in leaven samples after 14 days of fermentation was carried out using the high-performance liquid chromatography method. Agilent Technologies 1200 series liquid chromatograph was equipped with an autosampler (G1329B), pump (G1312B), refractic index detector (G1362A), and a diode array detector (G1315C) with a spectrum survey (190–400 nm) at a wavelength of 210 nm (Aligent Technologies, Santa Clara, CA, USA). A Rezex ROA 300 × 7.80 mm column (Phenomenex, Torrance, CA, USA) was used with isocratic elution consisting of 0.005 N H_2_SO_4_ at a flow rate of 0.6 mL/min. Measurements were carried out at a temperature of 40 °C. Samples were applied to the column in the amount of 10 µL. Qualitative and quantitative identification was performed using the external standard method (ChemStation for LC 3D systems, Aligent Technologies, Santa Clara, CA, USA).

### 2.6. Quantitative Analysis of Betalains in Leaven Samples

Quantification of betalains in leaven samples after 14 days of the fermentation process was carried out by using the Nilsson spectrophotometric method [9]. Firstly, 2 mL of the sample was diluted with 0.1 M phosphate buffer (pH = 6.5) to final absorbance in the range of 0.3 to 0.8 at 538 nm (Helios Delta spectrophotometer, ThermoFisher, Waltham, MA, USA). Then, the absorbance of the sample was measured at 476, 538 and 600 nm. The results were expressed in the equivalents of mg betanin/L (purple dye content) and mg vulgaxanthin/L (yellow dye content).

### 2.7. Statistical Analysis

All assays, except for the next-generation sequencing, were performed in triplicate. The results represent the mean values and the standard deviations. Statistical evaluation was based on Kruskal–Wallis and Mann–Whitney non-parametric tests at the significance level of *p* < 0.05 using STATISTICA 10.0 software (StatSoft, Kraków, Poland).

## 3. Results

### 3.1. Evaluation of the the Fermentation Process Kinteics

**Figure 1:**
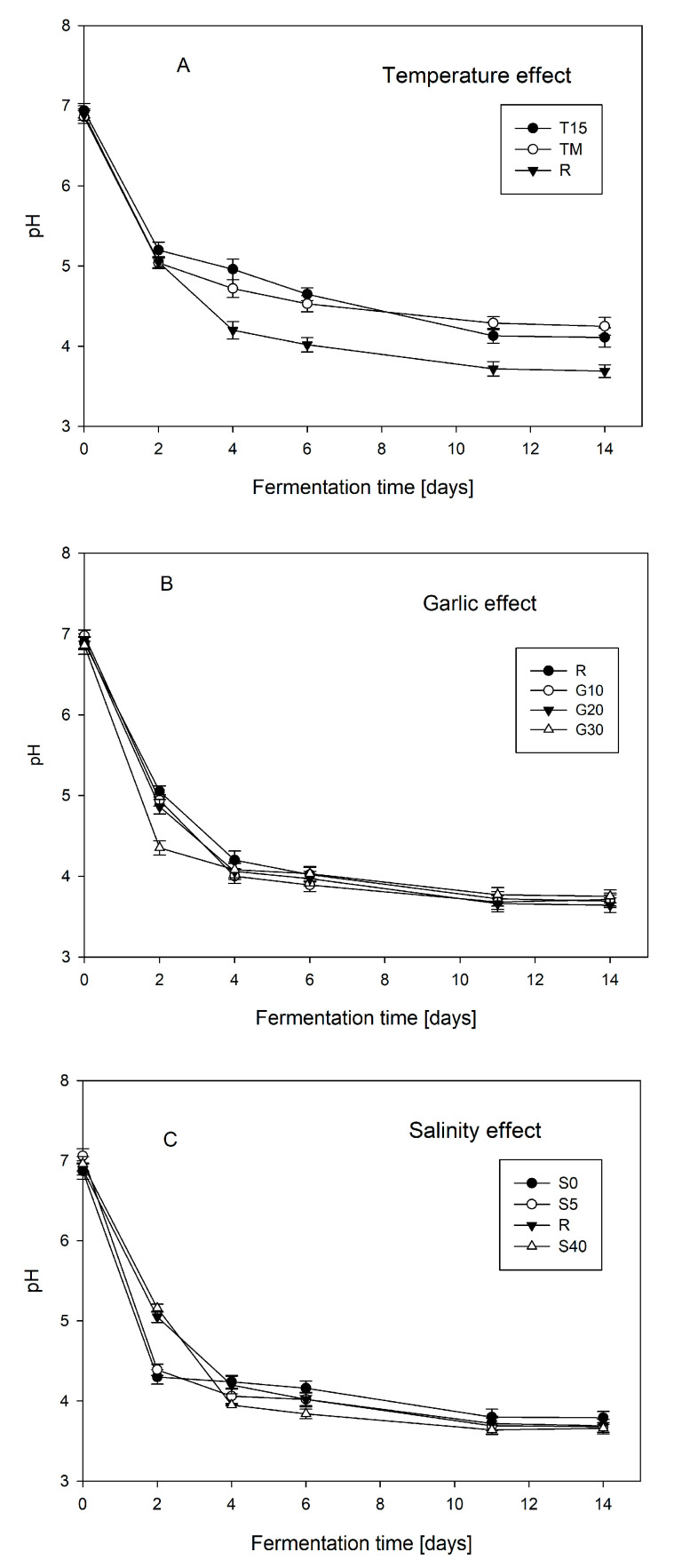
The effect of temperature (**A**), garlic dose (**B**), and brine salinity (**C**) on the pH decrease during beetroot fermentation. The symbols represent the following experimental variants: R—control; T15—temperature 15 °C; TM—temperature 25 °C for 48 h, and then followed by 15 °C; G10—garlic dose 10 g/jar; G20—garlic dose 20 g/jar; G30—garlic dose 30 g/jar; S0—salinity 0%; S5—salinity 5%; S40—salinity 40%.

Figure 1 shows the effect of temperature, brine salinity, and the garlic dose on the fermentation process expressed as a decrease in pH along the time course. There was no significant effect of temperature on the fermentation in the first 48 h of the process. From 96 h, a slowdown in the fermentation was observed in the lower temperature variant (T15). After 14 days of the process, a significantly lower decrease in pH was found in the low-temperature (T15) and variable temperature (TM) variants in relation to the reference sample (R). The pH values for these variants were 4.11 (T15), 4.25 (TM), and 3.69 (R), respectively.

Reducing the brine salinity (variants S0 and S5) contributed to a bigger decrease in the pH in the first 48 h of the process. No significant (*p* < 0.05) differences between variants containing lower NaCl concentrations: 0% (S0) and 0.5% (S5) and between variants containing higher NaCl concentrations: 2% (R) and 4% (S40) were detected. The final product did not show statistically significant (*p* < 0.05) differences in active acidity among analyzed variants. The addition of garlic (in variant G30) contributed to a significant increase in the fermentation in the first two days of the process. The lower content did not show a statistically significant (*p* < 0.05) effect on the fermentation process.

### 3.2. Quantitative Microbiological Analysis

**Figure 2:**
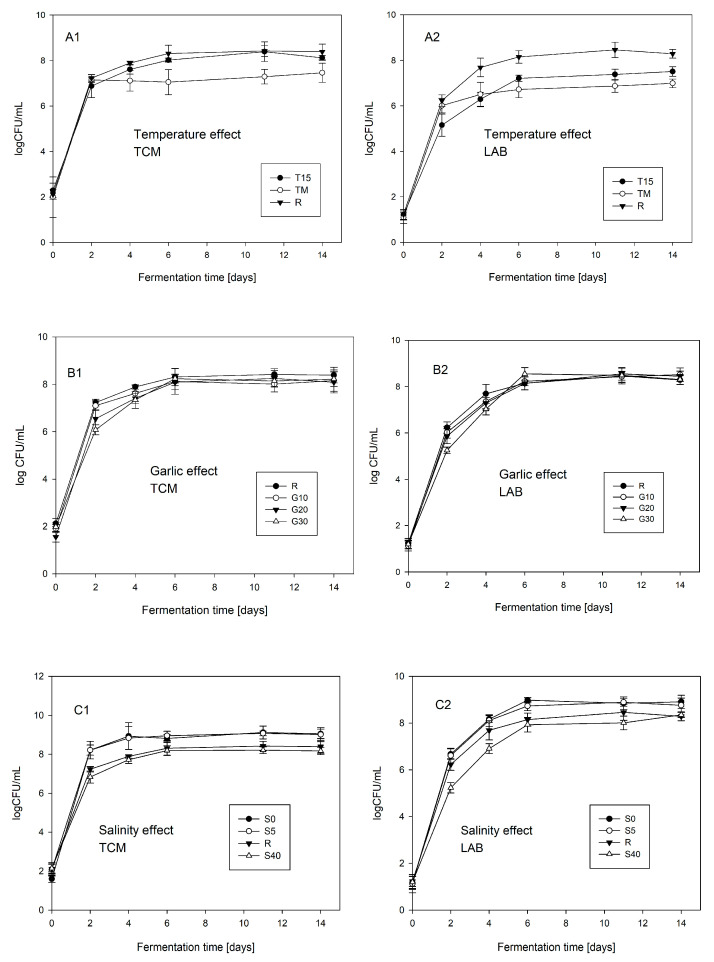
The effect of temperature (**A1**,**A2**), garlic dose (**B1**,**B2**), and brine salinity (**C1**,**C2**) on the number of culturable bacteria in beetroot fermentation systems. 1—number of culturable mesophilic bacteria; 2—number of culturable lactic acid bacteria. The symbols represent the following experimental variants: R—control; T15—temperature 15 °C; TM—temperature 25 °C for 48 h, and then followed by 15 °C; G10—garlic dose 10 g/jar; G20—garlic dose 20 g/jar; G30—garlic dose 30 g/jar; S0—salinity 0%; S5—salinity 5%; S40—salinity 40%.

The results of the quantitative assessment of the number of TCM and LAB in beetroot leaven during fermentation are presented in Figure 2.

A significant effect of temperature on the growth of both analyzed groups of microorganisms was observed. In the variant with a low fermentation temperature (T15), from 48 h, a reduced number of LAB was demonstrated compared the reference variant (R), but there were no significant differences between TCM throughout the fermentation process. In the variant with a variable temperature (TM), a clear, statistically significant (*p* < 0.05) inhibition of growth of both analyzed groups of microorganisms was observed from 48 h.

There was no statistically significant (*p* < 0.05) effect of garlic on the number of TCM and LAB in the range of the garlic concentrations used.

The statistically significant (*p* < 0.05) inhibitory effect of higher brine salinity (variants R and S40) compared to lower salinity (variants S0 and S5) on the growth of both analyzed groups of microorganisms in the first 96 h of fermentation was observed.

### 3.3. Analysis of the Organic Acids Profile

**Figure 3:**
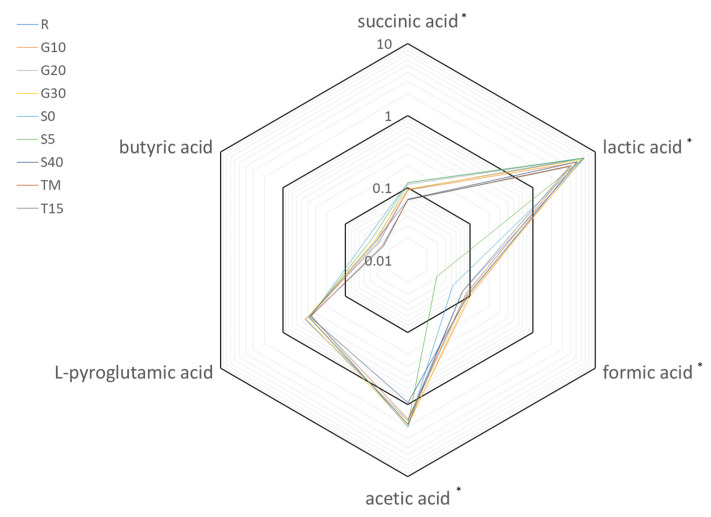
Radar graph summarizing the changes of organic acids concentration (mg/mL) in logarithmic scale in samples after 14 days of fermentation. Asterisks indicate significant differences among different samples (*p*-value < 0.05). The symbols represent the following experimental variants: R—control; T15—temperature 15 °C; TM—temperature 25 °C for 48 h, and then followed by 15 °C; G10—garlic dose 10 g/jar; G20—garlic dose 20 g/jar; G30—garlic dose 30 g/jar; S0—salinity 0%; S5—salinity 5%; S40—salinity 40%.

Analysis of the selected organic acids concentration in beetroot leaven proved the existence of similar patterns in all experimental variants (Figure 3). Lactic acid (values from 6.68 mg/mL to 3.87 mg/mL) and acetic acid (values from 0.95 mg/mL to 2.05 mg/mL) were simultaneously characterized by the highest concentration and highest variation of concentrations among the experimental variants. The lowest variation was observed for butyric acid (values from 0.02 mg/mL to 0.04 mg/mL). Moreover, for butyric acid and L-pyroglutamic acid, there were no statistically significant (*p* < 0.05) differences in the content between the experimental variants.

The NaCl content in brine did not significantly affect the concentration of lactic acid. In the highest brine salinity variant (S40), significantly less concentration of succinic acid (0.07 mg/mL, 27% lower than in reference) and acetic acid (0.95 mg/mL, 44% lower than in the reference) were observed. In the variants with a lower brine salinity (S0 and S5), a statistically significantly (*p* < 0.05) lower concentration of formic acid was detected (0.05 mg/mL for S0 and 0.03 mg/mL for S5, 30% and 61% lower than in reference, respectively).

In all experimental variants containing the addition of garlic, a significantly higher concentration of formic acid (0.09–0.10 mg/mL) compared to the reference sample (0.07 mg/mL) was detected. The concentration of other organic acids did not differ significantly from the reference sample.

In variants incubated in the low and variable temperatures (T15 and TM), significantly lower concentrations of lactic acid (3.87 mg/mL for TM and 4.19 mg/mL for T15) and succinic acid (0.07 mg/mL for both TM and T15) were noted. This represents a decrease of 37% (TM) and 42% (T15) for lactic acid and a decrease of 29% (TM and T15) for succinic acid compared to the reference sample. The concentration of the other organic acids did not differ statistically from the reference sample.

### 3.4. Quantitative Analysis of Betalains after the Fermentation Proces

The final product of the fermentation process was characterized by a relatively small, statistically insignificant (*p* < 0.05) difference in the content of violet and yellow dyes in most of the experimental variants in relation to the reference sample (R) (Table 2). A positive effect of the highest brine salinity variant (S40) on the content of both pigments’ analyzed dyes was only noted.

### 3.5. Analysis of the Bacterial Metapopulation Structure and Biodiversity

**Figure 4:**
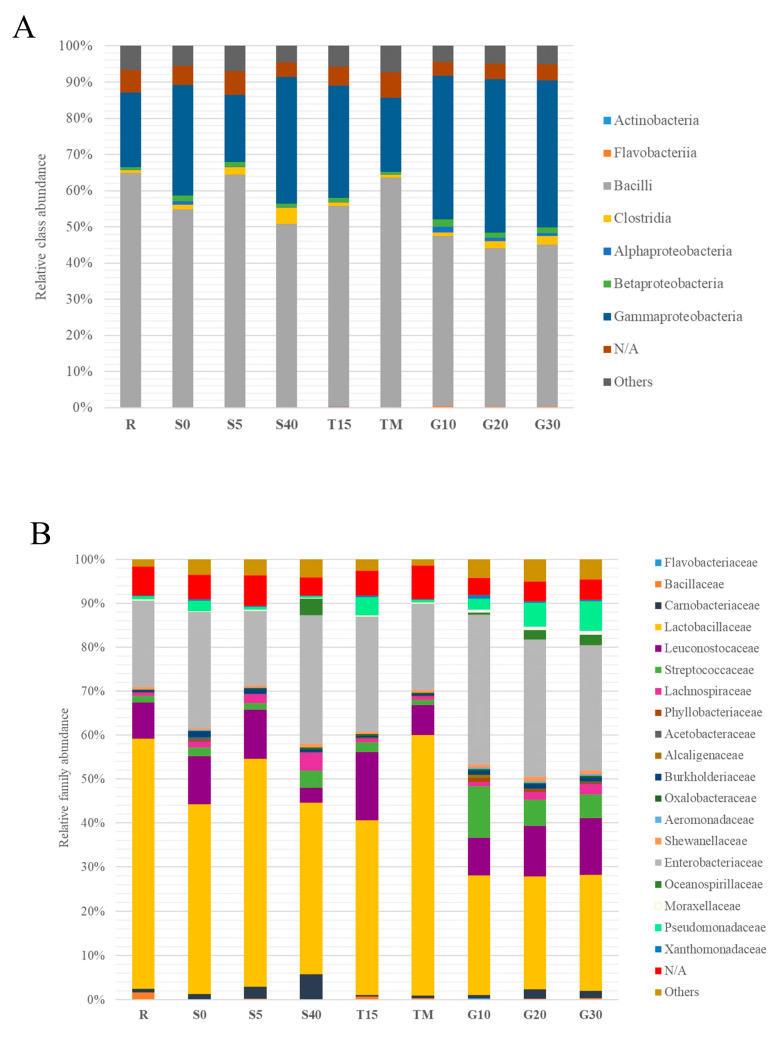
Relative abundance of bacteria at the class (**A**) and family (**B**) level in experimental variants after 14 days of fermentation. The symbols represent the following experimental variants: R—control; T15—temperature 15 °C; TM—temperature 25 °C for 48 h, and then followed by 15 °C; G10—garlic dose 10 g/jar; G20—garlic dose 20 g/jar; G30—garlic dose 30 g/jar; S0—salinity 0%; S5—salinity 5%; S40—salinity 40%.

Next-generation sequencing of bacterial 16S rRNA genes isolated from leaven samples after 14 days of fermentation allowed for the detection of a total of 10,994 OTUs, including 1626 species, 619 genus, 302 families, 165 orders, 68 classes, and 30 phyla. The relative abundance of major bacteria classes and families are shown in Figure 4. The differences can be observed between all experimental variants; however, classes *Gammaproteobacteria* and *Bacilli* and families *Lactobacillaceae*, *Leuconostocaceae,* and *Enterobacteriaceae* were dominant in all variants. Core microbiome analysis (sample prevalence > 10%, relative abundance > 0.01%) allowed to select the 31 of the most prevalent species, 23 most prevalent genus, and 12 most prevalent families (Supplementary Materials, Figures S1–S3). *Lactobacillaceae*, *Enterobacteriaceae,* and *Leuconostocaceae* were observed to be the most prevalent families among tested samples. The most prevalent genera were *Lactobacillus*, *Serratia*, *Leuconostoc,* and undefined, non-assigned taxa. The most prevalent species were *Lactobacillus plantarum*, *Lactobacillus brevis*, *Leuconostoc mesenteroides,* and two undefined, unassigned taxa.

In the variants containing garlic, an increase in the share of the *Gammaproteobacteria* class and a decrease in the share of the *Bacilli* class was noted, compared to the reference sample. Moreover, an increase in the share of *Streptococcaceae*, *Leuconostocaceae*, *Enterobacteriaceae,* and *Pseudomonadaceae* families and a decrease in the share of the *Lactobacillaceae* family was reported. A comparable trend (with the exception of the *Streptococcaceae* family) can be observed in the variant with lowered temperature (T15). In addition, there is a strong similarity between the variable temperature variant (TM) and the reference sample (R) at the level of classes and families.

The effect of brine concentration on the share of individual taxa was observed. Variants containing extreme values of salt concentration (S0 and S40) show a smaller share of the *Bacilli* class and a bigger share of the *Gammaproteobacteria* compared to the reference sample, as well as a smaller share of the *Lactobacillaceae* family and a bigger share of the *Pseudomonadaceae* family. The inhibitory effect of high salt concentration (S40) on the contribution of the family *Leuconostocaceae* was also detected. Moreover, in the S40 variant, the relatively high share (3.9%) of the *Oceanospirillaceae* family was observed.

In order to assess the potential contamination of silage with pathogenic bacteria, an analysis of the presence of selected taxa was carried out. These bacteria were mentioned in the literature as the most frequently detected human pathogens in fermented food: *Escherichia coli*, *Shigella* spp., *Salmonella* spp., enterotoxigenic *Staphylococcus aureus*, *Listeria monocytogenes*, and *Bacillus cereus* [10]. The results are presented in Supplementary Materials (Table S1). The presence of the above-mentioned taxa had a negligible contribution in all experimental variants (order of magnitude was 0.01% relative abundance).

**Figure 5:**
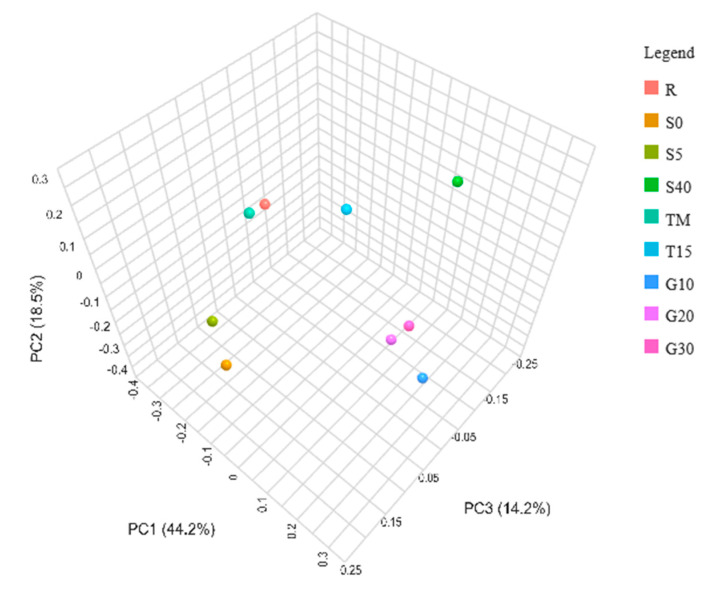
3D PCoA based on the Bray–Curtis distance matrix showing the β-diversity of bacteria communities among experimental variants after 14 days of fermentation. The symbols represent the following experimental variants: R—control; T15—temperature 15 °C; TM—temperature 25 °C for 48 h, and then followed by 15 °C; G10—garlic dose 10 g/jar; G20—garlic dose 20 g/jar; G30—garlic dose 30 g/jar; S0—salinity 0%; S5—salinity 5%; S40—salinity 40%.

**Figure 6:**
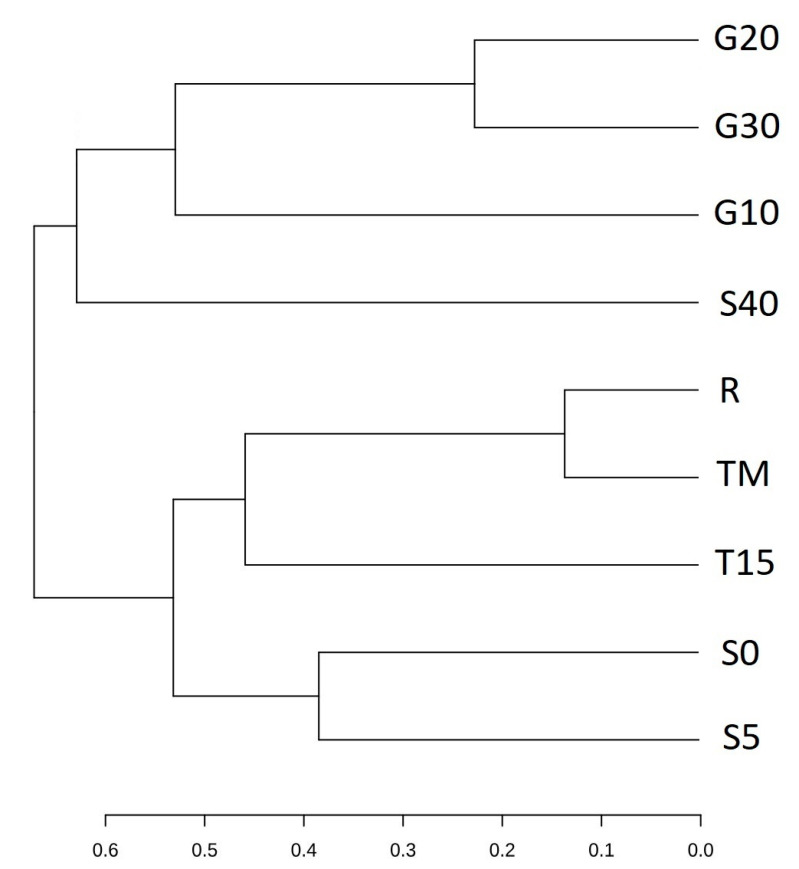
Dendrogram based on the values of Jaccard index presenting the similarity of bacteria populations in experimental variants after 14 days of fermentation. The symbols represent the following experimental variants: R—control; T15—temperature 15 °C; TM—temperature 25 °C for 48 h, and then followed by 15 °C; G10—garlic dose 10 g/jar; G20—garlic dose 20 g/jar; G30—garlic dose 30 g/jar; S0—salinity 0%; S5—salinity 5%; S40—salinity 40%.

The similarity of the metapopulation structure among the tested samples was presented using principal coordinate analysis (PCoA) and dendrogram clustering (Figure 5 and Figure 6). Several groups with a high level of similarity can be noted: (1) variants containing garlic (G10, G20, and G30), (2) variants containing low salt concentration (S0 and S5), (3) the reference variant (R) and the variant of varied incubation temperature (TM). The variant of low incubation temperature (T15) and the variant of high salt concentration (S40) show the smallest similarity to the other samples.

To assess biodiversity, the Chao1-bias corrected (richness) and Simpson index (evenness) coefficients were determined (Table 3). Samples with lower salt concentrations (S0 and S5) were characterized by a higher species richness. A similar relationship was observed in variants with lower incubation temperature (T15), varied temperature (TM), and with the addition of garlic in doses of 20 g (G20) and 30 g (G30).

## 4. Discussion

Red beet (*Beta vulgaris* L.) is considered to be a source of important compounds such as phenolic compounds and betalains, which have a wide range of health benefits: they counteract oxidative stress, possess antibacterial and antiviral properties, and play an important role in the dietary prevention of cardiovascular and cancer diseases [11,12,13]. The consumption of beetroot leaven, apart from the benefits associated with a high phenolic compounds content, can introduce probiotic lactic acid bacteria, which improve gut health and decrease hyperglycemia [14]. Although it is believed that traditional fermented products are characterized by a large diversity due to the multitude of intrinsic and extrinsic factors that may affect the course of the microbial bioconversion of raw materials [15,16], these studies have shown that regardless of the variable addition of garlic, salinity, and fermentation temperature, the analyzed red beet leaven exhibit a high pro-health potential both in the context of the concentration of betalains and the lactic acid bacteria content.

This study confirmed that there is no significant effect of the fermentation temperature and the garlic addition on the content of betacyanins and betaxanthins in the beetroot leaven after 14 days of fermentation. As suggested by Sawicki and Wiczkowski (2018), the concentration of betalain dyes during the fermentation is the result of the coexistence of two processes: the releasing of dyes into the brine from beetroot tissue (as a result of its maceration under the influence of microorganisms), and the biodegradation and biotransformation of dyes by fermenting microorganisms [17]. It can be assumed that differences in the taxonomic composition of the microbiota, changes in their activity, and abundance in the analyzed variants did not affect the content of dyes in the final products due to the coexistence of two antagonistic processes: dye releasing from beetroot tissue and dye degradation. Additionally, betalains have shown broad pH stability (ranged from 3 to 7) and temperature stability below 50 °C [18], which makes them resistant to non-microbial degradation in unpasteurized beetroot leaven. A positive relation between the high salinity and the content of betacyanins and betaxanthines can be explained by the action of osmotic forces that increased the efficiency of dyes released from beetroot tissue to the solution.

It is believed that fermented products can be considered as a promising source of probiotic microorganisms and defined as probiotic food if they contain 6.00–8.00 Log CFU/g [3,19]. Despite the significantly lower number of culturable lactic acid bacteria observed in variants with lower and variable fermentation temperature (TM and T15)—all variants of leaven meet the above criterion and can be defined as a potential source of microorganisms with probiotic properties.

Despite the use of vegetables from organic farming and maintaining low hygienic standards (only preliminary washing and no peeling), no significant risk of foodborne pathogens was found. The number of DNA copies of potentially pathogenic strains in the final product was relatively negligible, within the margin of sequencing error. This is probably due to the antagonistic effect of the lactic acid bacteria (LAB) against the pathogenic microbial strains. The antimicrobial activity of LAB has been previously confirmed towards *Salmonella* spp., *Listeria monocytogenes,* and *Escherichia coli*. The antagonistic effect of LAB is associated with many mechanisms of interaction, and it is well described in the literature [20,21,22]. The most important seems to be the competitive interaction by modifying the ecological niche through the synthesis of organic acids and lowering the pH, as well as the synthesis of bacteriocins [23].

The temperature, salinity, and garlic addition impact the formation of the selected organic acids content in the beetroot leaven. Shaping the organic acid profile can be associated with the differences in beetroot leaven metabiome composition, and thus, with the diversity in metabolic potential among tested variants. The importance of the metabiome’s composition for determination of organoleptic properties in fermented products has been confirmed in previous studies [24,25,26]. It can be concluded that the red beet leaven microbiome is highly sensitive to physicochemical factors. The process of shaping the microbiota and determining the dominance between taxa during traditional fermentation is a directed process, which was confirmed by the analysis of β-biodiversity of leaven variants. The impact of the individual analyzed factors is discussed in detail below.

The importance of salinity

Brine salinity is considered to be one of the most important factors affecting the food fermentation process, the metabolic potential of microorganisms, and the sensory profile of the final product [27]. The results of quantitative analyses of LAB and TCM indicate a presence of a transitional period of bacterial population adaptation. The initial, partial inhibition of the fermentation process may also result from the slowdown in the metabolism of bacteria, including LAB, and delayed entry into the logarithmic growth phase. This effect was also observed in earlier studies on fermented sauerkraut [28]. The positive impact of the high salinity (40 g/L) on the share of the *Oceanospirillaceae* can be explained by the fact that this family includes halophilic microorganisms [29]. The decline in species richness indicates the existence of targeted changes favoring the growth and dominance of taxonomically close microbial groups. Salinity has a large impact on the balance among the share of dominant taxa. The positive effect of the increase in salinity from 0% to 2% on the share of the *Lactobacillaceae* family and the decrease in its share due to the higher salinity of 4% suggests that there is an optimal salinity between 2% and 4% for this group of microorganisms. Rao et al. (2004) indicate the salinity of 4% as optimal for the growth and activity of the *Lactobacillaceae* family members [30]. However, this study did not take into account the importance of intra-population factors, such as antagonistic interactions. The results of this study suggest the existence of antagonistic interactions between members of the *Lactobacillaceae* and *Enterobacteriaceae* families. The antagonistic nature of interactions between representatives of these groups of microorganisms has been previously described in the literature [31,32]. The directed effect of salinity is also visible in the case of the abundance of the *Leuconostocaceae* family; an increase in salinity results in a decrease in the share of this taxon. Representatives of this group (e.g., *Leuconostoc citreum*) can be considered as playing an important role in the fermentation of beetroot in the environment of reduced salt content, which was also suggested in another study concerning other fermented products [33]. Assuming the criterion of the maximum share of potentially probiotic bacteria and the minimum share of the family of potentially pathogenic microorganisms (*Enterobacteriaceae*), brine concentration in the range of 0.5–2% can be suggested as the most favorable.

The importance of garlic

Aqueous garlic extracts are a rich source of organosulfur compounds such as allicin, ajoene, and various aliphatic sulfides, whose antimicrobial properties have been well described in the literature [34]. Despite the presumed antimicrobial properties, no negative effect of this additive on the TCM and LAB growth was found, which can be associated with a relatively low extraction efficiency and low bioavailability of antimicrobial compounds mentioned above. On the other hand, the significant change in the metapopulation structure of beetroot leaven found in all samples with the garlic addition indicates the existence of a factor influencing the balance between taxonomic groups in the metapopulation. These changes were probably caused by the biotic factor, which was the autochthonous microbiota of garlic bulbs. An example supporting this thesis are higher values of all alpha biodiversity indicators in variants containing garlic and the increase in the share of *Pseudomonaceae* and *Enterobacteriaceae* families. These taxa were claimed as dominant in the microbiota of garlic bulb cloves [35]. Targeted changes in taxonomic structure and thus changes in overall metabolic metapopulation potential probably affect the formic acid fermentation pathways. Taking into account only the microbiological evaluation criterion mentioned in the previous paragraph, excluding the possible impact of garlic on sensory properties, its addition in a non-sterilized and unpeeled form is not beneficial for the quality of the final product.

The importance of fermentation temperature

It is believed that the process of lactic fermentation of raw materials of plant origin includes several stages, among which the first phase seems to be the most important. During this phase, facultatively anaerobic microorganisms synthesize carbon dioxide and create conditions for the development of other anaerobic microorganisms, which play a dominant role in the subsequent phases [36]. Lowering the temperature (at the beginning or after 48 h of the process) probably resulted in slowing down the metabolism of microorganisms and a delay in the formation of anaerobic conditions. This was associated with a slower fermentation at further stages, a higher pH of the leaven, and a lower concentration of selected acids (formic acid and lactic acid). Based on the analysis of the growth of bacteria, it can be assumed that bacteria with a wide temperature tolerance are present on the red beet roots. However, they do not include LAB, for which the optimum activity and growth is mostly in the range of higher temperatures of 20–30 °C [37]. The first 48 h of the fermentation process were crucial in shaping the bacterial population and functional structure. Lowering the temperature at the beginning of the fermentation process probably contributed to a more effective growth and dominance of taxa with a wide range of temperature tolerance and an increase in the share of representatives of the *Pseudomonadaceae*, *Enterobacteriaceae,* and *Leuconostocaceae* families. Lowering the temperature after 48 h of the process did not significantly affect the metapopulation composition and the share of individual taxa, but had a negative impact on the final quality of the leaven. Therefore, conducting fermentation at constant, relatively high temperatures (20 °C) seems to be the most optimal.

## 5. Conclusions

The conducted research allowed for partial confirmation of the hypotheses. It has been proven that the brine salinity, temperature, and garlic addition significantly affect the spontaneous red beet fermentation process, in particular in the context of the qualitative composition of the microbiota and the profile of organic acids. It has also been proven that these changes are targeted and thus potentially controllable. The variable factors mentioned above do not affect the status of the product as a potential source of probiotic bacteria. The concentration of sodium chloride in brine has a positive effect on the concentration of betalains in the beetroot leaven; however, the increase of this bioactive compound content should be confronted with possible negative aspects associated with the consumption of large amounts of salt. The addition of the unpeeled form of garlic and lowering the fermentation temperature to 15 °C were not beneficial for the leaven quality. Research shows that the use of beetroot from organic farming along with minimized hygienic standards, regardless of the tested factors, is associated with a low risk of the presence of pathogenic microorganisms in the final product and allows to obtain a fermented product with high health-promoting properties. However, as food-borne pathogens are a serious concern, the careful washing of beetroots with tap water before fermentation is always recommended.

## Figures and Tables

**Table 1:** Characteristics of red beet fermentation variants.

Symbol of Variant	Analyzed Factor	Characteristics of Variant		
		Brine Salinity	Temperature	Garlic Addition
R	Reference	20 [gNaCl/L]	25 °C	0 g
T15	Temperature	20 [gNaCl/L]	15 °C	0 g
TM		20 [gNaCl/L]	25 °C for 48 h, and then 15 °C	0 g
G10	Garlic	20 [gNaCl/L]	25 °C	10 g
G20		20 [gNaCl/L]	25 °C	20 g
G30		20 [gNaCl/L]	25 °C	30 g
S0	Brine salinity	0 [gNaCl/L]	25 °C	0 g
S5		5 [gNaCl/L]	25 °C	0 g
S40		40 [gNaCl/L]	25 °C	0 g

**Table 2:** The content of betacyanin and betaxanthins pigments in leaven samples. The symbols represent the following experimental variants: R—control; T15—temperature 15 °C; TM—temperature 25 °C for 48 h, and then followed by 15 °C; G10—garlic dose 10 g/jar; G20—garlic dose 20 g/jar; G30—garlic dose 30 g/jar; S0—salinity 0%; S5—salinity 5%; S40—salinity 40%.

Variant Name	The Content of Violet Dyes—Betacyanin [mg Betanin/L]	Content of Yellow Pigments—Betaxanthins [mg Vulgaxanthines/L]
R	88 ± 12	15 ± 2
G10	95 ± 11	14 ± 1
G20	102 ± 13	15 ± 1
G30	93 ± 9	16 ± 1
TM	89 ± 6	14 ± 2
T15	92 ± 9	15 ± 2
S0	67 ± 13	12 ± 3
S5	74 ± 13	13 ± 2
S40	125 ± 12	18 ± 1

**Table 3:** Bacterial community alpha diversity indices in leaven samples after 14 days of fermentation. The symbols represent the following experimental variants: R—control; T15—temperature 15 °C; TM—temperature 25 °C for 48 h, and then followed by 15 °C; G10—garlic dose 10 g/jar; G20—garlic dose 20 g/jar; G30—garlic dose 30 g/jar; S0—salinity 0%; S5—salinity 5%; S40—salinity 40%.

	R	S0	S5	S40	TM	T15	G10	G20	G30
Chao1 bias-corrected	1523	1923	2121	1417	1811	2048	1489	1941	1899
Simpson index	0.95	0.97	0.96	0.97	0.95	0.97	0.97	0.99	0.98

## Data Availability

Raw sequencing output data have been deposited at the National Center for Biotechnology Information (SRA repository), as BioProject under ID PRJNA980946. The other data that support the findings of this study are available from the corresponding author upon request.

## Supplementary Materials

The following supporting information can be downloaded at: https://www.mdpi.com/article/10.3390/foods12163079/s1, Figure S1: Heatmap presenting the core microbiome prevalence in all experimental variants at bacterial species level; Figure S2: Heatmap presenting the core microbiome prevalence in all experimental variants at bacterial genus level; Figure S3: Heatmap presenting the core microbiome prevalence in all experimental variants at bacterial family level; Table S1: The number of reads of potential human pathogens obtained by the sequencing at the species level.
