# Supplementary material for: Temperature, Salinity and Garlic Additive Shape the Microbial Community during Traditional Beetroot Fermentation Process
Source: Foods. 2023 Aug 16;12(16):3079. doi: 10.3390/foods12163079 (PMC10453225; doi:10.3390/foods12163079)
Supplement: Supplementary file 1 [file foods-12-03079-s001.zip › Table S1.pdf]

Supplementary table

Table S1. The number of reads of potential human pathogens obtained by the sequencing at the species level

Species	R	S0	S5	S40	T15	TM	G10	G20	G30
Salmonella sp. DAP8	n/d	n/d	3	n/d	n/d	2	2	n/d	n/d
Uncultured Salmonella sp	n/d	n/d	n/d	n/d	n/d	n/d	50	n/d	2
Salmonella enterica subsp. enterica serovar Typhi	n/d	n/d	n/d	n/d	n/d	n/d	9	n/d	2
Salmonella enterica subsp. enterica serovar Typhimurium	n/d	n/d	n/d	n/d	n/d	n/d	n/d	n/d	n/d
Salmonella enterica subsp. houtenae str. ATCC BAA-1581	n/d	n/d	n/d	n/d	n/d	n/d	n/d	2	3
Salmonella sp. Co9936	n/d	n/d	n/d	2	n/d	n/d	n/d	2	n/d
Salmonella enterica	n/d	n/d	n/d	n/d	n/d	n/d	10	n/d	n/d
Escherichia coli	21	11	21	14	11	14	n/d	8	8
Escherichia sp. 4B	n/d	n/d	n/d	2	n/d	n/d	2	n/d	n/d
Uncultured Escherichia sp	n/d	n/d	n/d	n/d	n/d	n/d	n/d	n/d	2
Uncultured Shigella sp	n/d	n/d	n/d	n/d	n/d	n/d	2	n/d	2
Staphylococcus aureus	4	2	5	6	4	2	n/d	4	1
Listeria monocytogenes	2	n/d	n/d	2	n/d	2	n/d	n/d	n/d
Bacillus cereus	n/d	3	n/d	n/d	n/d	n/d	n/d	n/d	n/d
