# Supplementary figures and images for: Temperature, Salinity and Garlic Additive Shape the Microbial Community during Traditional Beetroot Fermentation Process
Source: Foods. 2023 Aug 16;12(16):3079. doi: 10.3390/foods12163079 (PMC10453225; doi:10.3390/foods12163079)

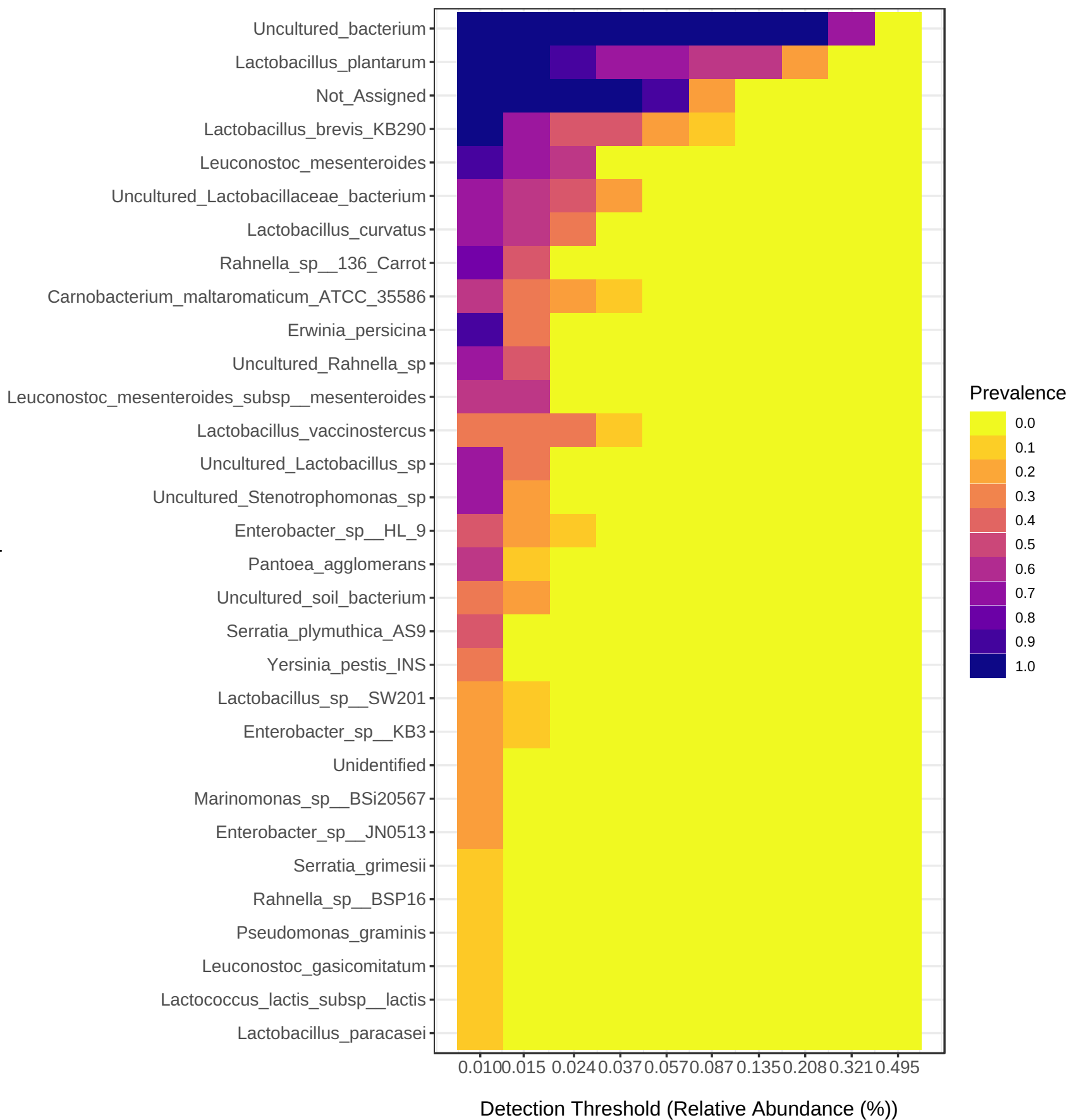

Supplement: Supplementary file 1 [file foods-12-03079-s001.zip › Figure S1.pdf]

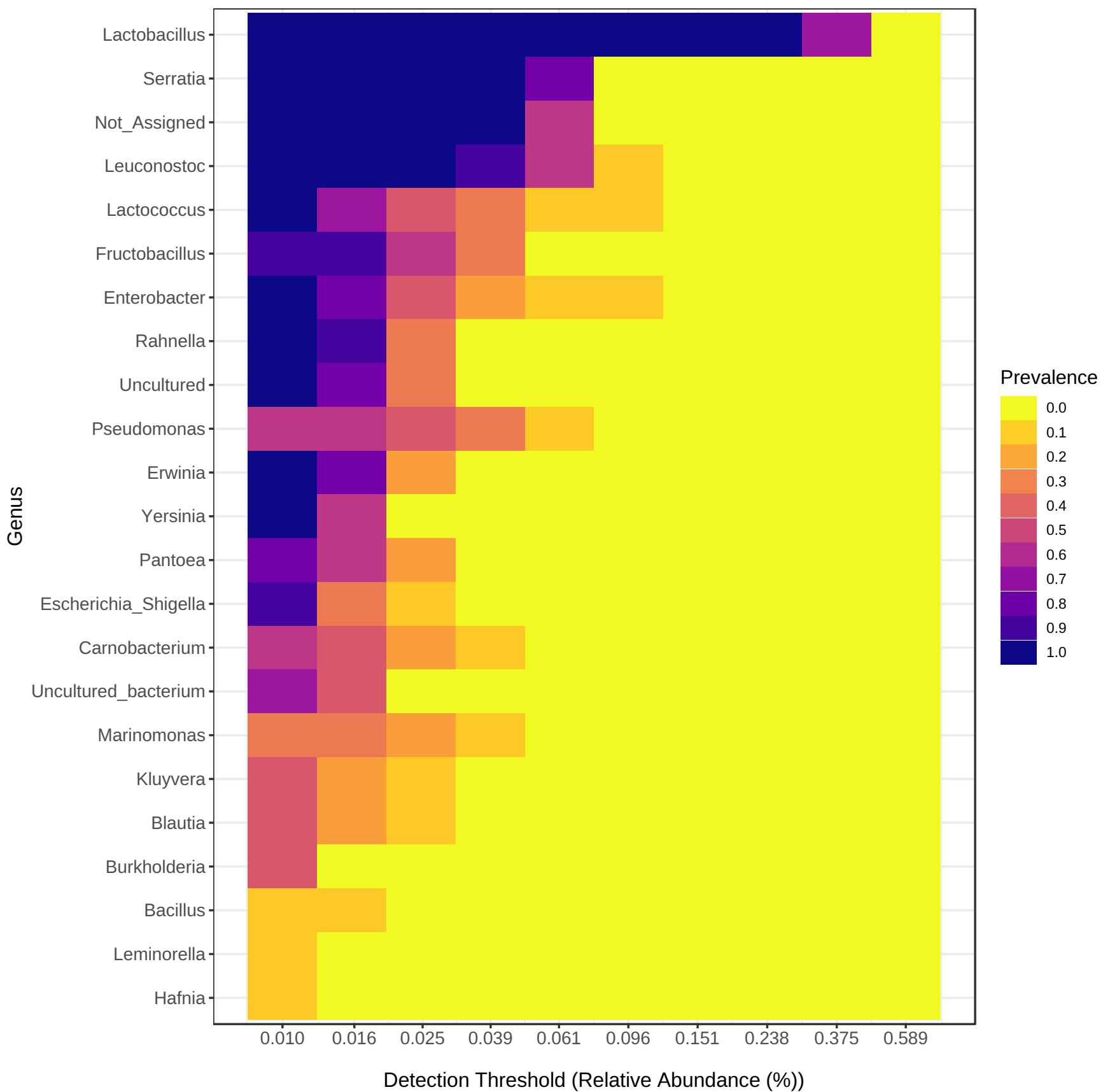

Supplement: Supplementary file 1 [file foods-12-03079-s001.zip › Figure S2.pdf]

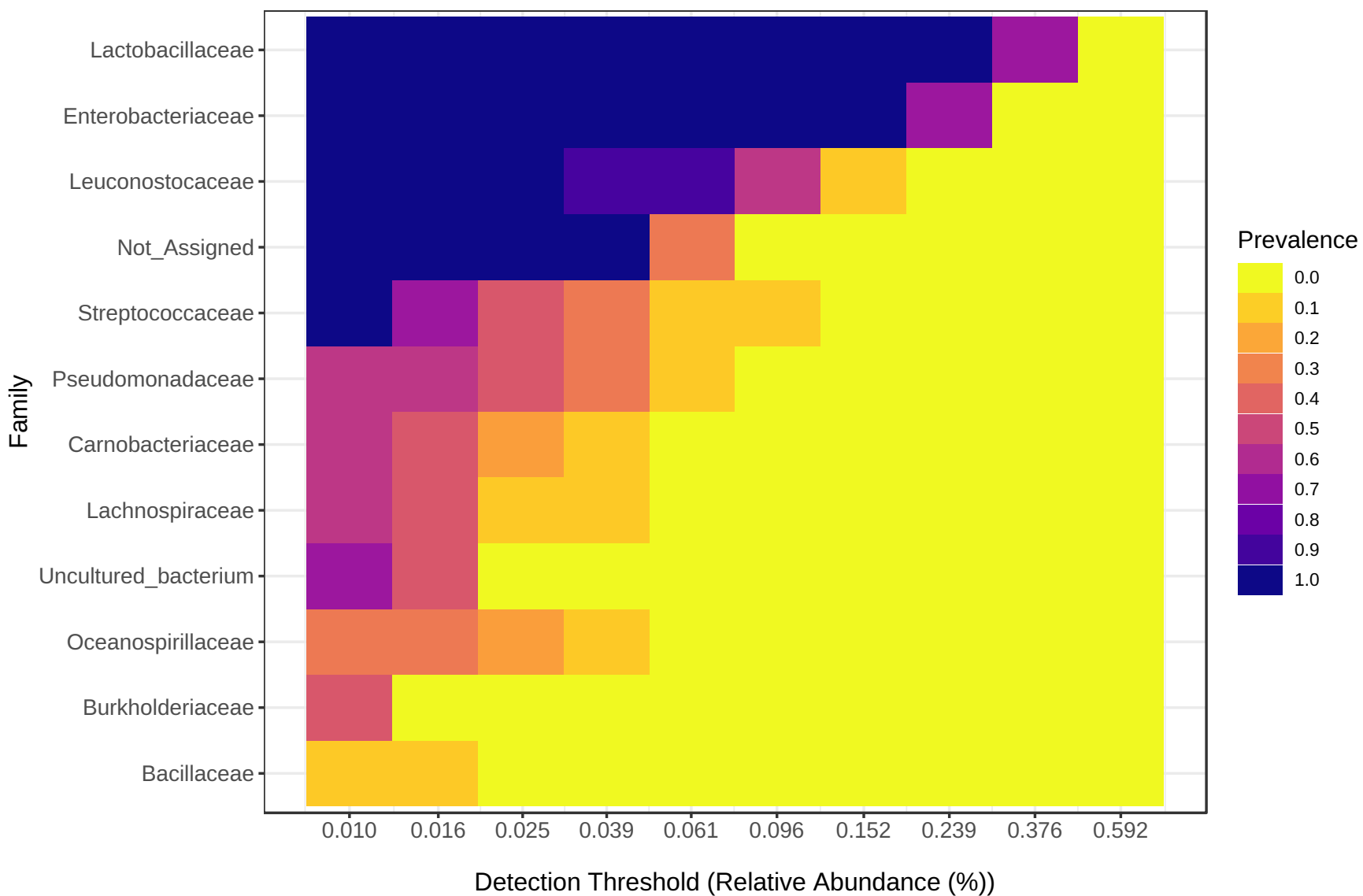

Supplement: Supplementary file 1 [file foods-12-03079-s001.zip › Figure S3.pdf]
